# Supplementary material for: Knowledge, understanding and perceptions of key stakeholders on the maternity protection available and accessible to female domestic workers in South Africa
Source: PLOS Glob Public Health. 2023 Jun 14;3(6):e0001199. doi: 10.1371/journal.pgph.0001199 (PMC10266589; doi:10.1371/journal.pgph.0001199)
Supplement: S1 Text — (DOCX) [file pgph.0001199.s001.docx]

**Interview guide for Individual Semi-structured Interviews with Key Stakeholders**

**Purpose of interview:** to gather information regarding key stakeholders’ knowledge and understanding of maternity protection benefits for female non-standard workers, to describe current maternity protection benefits that are supposed to be available to non-standard workers (particularly domestic workers) in the Western Cape/South Africa.

Date: _________________________

**Key Informant Description (demographic variables)**

Sector: _______________

Type / Name of Organization: _______________

Department Within Organization: _______________

Position Within Organization: _______________

Sex: ________________

Race: ________________

Hi, my name is XXXX. Thank you for taking the time to speak to me. I am doing my PhD in through the University of XXXX. The overall aim of my PhD research is “To examine current maternity health and economic protection benefits available and accessible to non-standard employee domestic workers, in the Western Cape, to improve understanding of potential implications for breastfeeding practices”.

*You can play this by ear depending on who you interview, unlike in academic writing, you may need to put this a bit more colloquially, for e.g.*

“In my PhD research I am aim to examine what existing maternity health and economic benefits are available to workers such as domestic workers in the Western Cape. I am particularly interested in knowing more about whether they are able to access the benefits that do exist and if has any effect on whether they are able to continue breast feeding their babies if they go back to work”. *Or something similar to this.*

As part of my research, I would like to ask you a few questions. The reason I have asked to speak to you is because of your position at XXXX or that XXXX recommended you because of your position at XXXX, to be able to answer a few questions regarding maternity protection benefits in South Africa. Your knowledge and experience can help me to understand a bit about the availability and accessibility of maternity protection for non-standard workers (especially domestic workers) in South Africa.

1. Firstly, in my research, I am using the term “maternity protection benefits”. What is your understanding of what comprehensive “maternity protection benefits” refer to? What benefits do you think that women should be able to access in South Africa, when they are working and then have a baby? (*Prompt/Probe: Can you list the benefits that women should be able to receive when they are pregnant and following the birth of a child?*)
2. Can you tell me about (name and describe) any specific policies or legislation (laws) you are aware of that exist to ensure that women are able to access maternity protection?
3. What role do you believe/think you have in ensuring that women are able to access maternity protection benefits?
4. Do you think that your department has a role to play in ensuring that women can access maternity protection benefits and if so, what is that role?
5. What do you think is the general understanding and awareness with regard to maternity protection benefits that women should receive in your organisation?
6. What do you think the similarities and differences are with regard to maternity protection benefits that are supposed to be available for women who are employed in full-term, permanent positions compared to women employed in forms of non-standard employment (such as in part-time or contract positions - for example domestic workers)?
7. (*Prompt/probe: Do you think that the benefits should be the same or different? How so?*)
8. If you think of female domestic workers specifically, what maternity protection benefits do you think they are legally entitled to, currently in South Africa?
9. Can you describe how you think the maternity protection benefits that female domestic workers are supposed to receive by law are currently being implemented? (*Probe/prompt: Are female domestic workers accessing the maternity protection that they should be able to? If yes / no, why or why not?*)
10. What type of maternity protection benefits do you think that female domestic workers should be able to access, considering that they are a group with varying employment contracts and arrangements?
11. Based on your knowledge and experience, can you think of anything that could or should be done and if so by whom, to improve the access of maternity protection benefits by female domestic workers specifically, or do you feel that this is good as it is? (*Probe/prompt: Keep same as it? Improve the implementation of the existing policy or legislation? Keep policy as is? Update existing policy / legislation to include….? Keep involvement of Department of Labour? Involve other sectors such as….?)*

Thank you for the time you have taken to provide this interview, I really appreciate it. You are welcome to contact me if you have any questions or additional points you would like to make.
