# Supplementary material for: Knowledge, understanding and perceptions of key stakeholders on the maternity protection available and accessible to female domestic workers in South Africa
Source: PLOS Glob Public Health. 2023 Jun 14;3(6):e0001199. doi: 10.1371/journal.pgph.0001199 (PMC10266589; doi:10.1371/journal.pgph.0001199)
Supplement: Attachment — Submitted filename: Responses to Editor and Reviewer comments_2Apr2023.docx [file pgph.0001199.s002.docx]

March 2023

PLOS Global Public Health: PGPH-D-22-01467

Knowledge, understanding and perceptions of key stakeholders on the maternity protection available and accessible to female domestic workers in South Africa

**Author responses to Editor and Reviewers’ comments received 7 March 2023**

| **Journal requirements** | **Authors’ responses** |
| --- | --- |
| 1. Please review your reference list to ensure that it is complete and correct. If you have cited papers that have been retracted, please include the rationale for doing so in the manuscript text, or remove these references and replace them with relevant current references. Any changes to the reference list should be mentioned in the rebuttal letter that accompanies your revised manuscript. If you need to cite a retracted article, indicate the article’s retracted status in the References list and also include a citation and full reference for the retraction notice. | We have not cited any papers that have been retracted. For reference 17, the weblink provided previously has stopped working, so we have inserted the revised weblink and this document is now publicly available for access. Pg 19. |
| 2. Please provide separate figure files in .tif or .eps format only and remove any figures embedded in your manuscript file. Please also ensure that all files are under our size limit of 10MB.  For more information about figure files please see our guidelines:  https://journals.plos.org/globalpublichealth/s/figures  https://journals.plos.org/globalpublichealth/s/figures#loc-file-requirement | Box 1 has been renamed as a figure and converted to .tif format file and we have inserted the caption for the figure at the end of the manuscript. Pg 18. |
| 3. We have noticed that you have uploaded Supporting Information files, but you have not included a list of legends. Please add a full list of legends for your Supporting Information files after the references list. | Have done so at the end of the manuscript. Pg 18. |
| 4. In the online submission form, you indicated that "The datasets generated and/or analysed during the current study are not publicly available to protect participant confidentiality but are available in an anonymised form from the corresponding author on reasonable request". All PLOS journals now require all data underlying the findings described in their manuscript to be freely available to other researchers, either 1. In a public repository, 2. Within the manuscript itself, or 3. Uploaded as supplementary information.  This policy applies to all data except where public deposition would breach compliance with the protocol approved by your research ethics board. If your data cannot be made publicly available for ethical or legal reasons (e.g., public availability would compromise patient privacy), please explain your reasons by return email and your exemption request will be escalated to the editor for approval. Your exemption request will be handled independently and will not hold up the peer review process, but will need to be resolved should your manuscript be accepted for publication. One of the Editorial team will then be in touch if there are any issues. | All data relevant to the manuscript has been presented within the manuscript itself (in the form of participant quotations). |

**Reviewer 1 comments**

| **Reviewer’s comments** | **Authors’ responses** |
| --- | --- |
| Thank you for the opportunity to review this manuscript. I was really intrigued with this study and found it very interesting. The paper is well-written and easy to follow.  The authors provide adequate background on the issue of domestic workers and their unique vulnerabilities in terms of health and well-being. I also appreciated the background on efforts to improve maternity protection globally and in SA. This contextual background helped me to better understand the results, and were also useful in justifying the importance of this study.  I think more insight into domestic workers’ health status makes an important contribution to the literature.  The methods section included mention of consent, and IRB approval. The authors explained the methods used for data protection. I also thought it was good to mention that the interviews were audio-recorded.  Interviews were conducted with key stakeholders until saturation point was reached. I think a snowball approach with purposive sampling made sense for this type of study. | Thank you very much for the feedback. |
| Since the interviews were done with stakeholders (who I presume were educated), I wonder why only verbal consent was required. | The interviews were conducted virtually on an online platform, and this made it difficult and time -consuming to insist that participants provided written consent (which may have required printing out, signing and scanning and then emailing a document back). as well as verbal consent |
| Also, I would like to know how the interview guide was developed and how many questions were included. What were the domains or types of questions asked? | The interview guide is provided as Supplementary Material (and might not have been seen by reviewers in the review process). It contains 11 guiding questions with some containing additional prompts and probes.  Have added the following sentence to the Data Collection sub-section of the Methods to describe how the interview guide was developed:  “Guiding questions were developed and adapted from sample interview guides available in the ILO Maternity Protection Resource Package (22).” – Pg 4 |
| I would also like to know more about the person who was the interviewer. What was this person’s training in qualitative research? Is this person a citizen of SA? | Have added that the interviewer was “a South African female PhD student trained in qualitative research”. (Pg 4) |
| I would also like to know if anyone else helped with coding. If not, that would seem like a major limitation of this study because it would be based on one person’s interpretation of the data. | Have added the following to the Data Analysis section:  “CPK coded the data and TD/MF read samples of the interview transcripts to confirm themes.” (Pg 4) |
| Results: it would be helpful to know how many when the authors say words like “some stakeholders” or “many stakeholders.” | In qualitative research, it is not appropriate to assign numerical values when describing responses. However, throughout the results section, we have edited the wording to be more descriptive and replaced the term “many stakeholders” with “almost all stakeholders” and “some stakeholders” with “several stakeholders” or “one participant” – where appropriate and in some instances we have now included which sector the participants were from (especially if it was one or only a few participants that indicated a certain issue). |
| It would also be helpful to know more about the stakeholders when citing examples or direct quotes. For example, is this person male or female, from the national or provincial pool, etc., instead of just using terms like (S10). | Thank you for this comment. Throughout the Results section, we have now added in the participant sex, and which sector they are from in place of the codes that were previously provided. |
| I thought the results had some important implications in terms of providing better protections to pregnant migrant workers. Obviously there was a lot of information about benefits that was not being disseminated. If these stakeholders were not familiar with some of the protective policies, it is even less likely that workers will know about them.  I thought the authors did a good job of linking the results to other previous studies. I also liked the recommendations to improve the maternal health of domestic workers. | Thank you very much for the feedback. |
